# Effects of Antiplatelet Drugs on Platelet-Dependent Coagulation Reactions

**DOI:** 10.3390/biom13071124

**Published:** 2023-07-14

**Authors:** Ivan A. Muravlev, Anatoly B. Dobrovolsky, Olga A. Antonova, Svetlana G. Khaspekova, Amina K. Alieva, Dmitry V. Pevzner, Alexey V. Mazurov

**Affiliations:** Chazov National Medical Research Center of Cardiology, Russian Ministry of Health, Academician Chazov Str., 15a, Moscow 121552, Russia; ivan_muravlev@mail.ru (I.A.M.); abdobrovolsky@inbox.ru (A.B.D.); loa_lu@mail.ru (O.A.A.); svkh@list.ru (S.G.K.); amina_alieva_1998@mail.ru (A.K.A.); pevsner@mail.ru (D.V.P.)

**Keywords:** platelets, blood coagulation, fibrin, thrombin generation, antiplatelet drugs, acetylsalicylic acid, ticagrelor, glycoprotein IIb-IIIa antagonists, prostaglandin E1

## Abstract

Activated platelets are involved in blood coagulation by exposing phosphatidylserine (PS), which serves as a substrate for assembling coagulation complexes. Platelets accelerate fibrin formation and thrombin generation, two final reactions of the coagulation cascade. We investigated the effects of antiplatelet drugs on platelet impact in these reactions and platelet ability to expose PS. Washed human platelets were incubated with acetylsalicylic acid (ASA), ticagrelor, ASA in combination with ticagrelor, ruciromab (glycoprotein IIb-IIIa antagonist), or prostaglandin E1 (PGE1). Platelets were not activated or activated by collagen and sedimented in multiwell plates, and plasma was added after supernatant removal. Fibrin formation (clotting) was monitored in a recalcification assay by light absorbance and thrombin generation in a fluorogenic test. PS exposure was assessed by annexin V staining using flow cytometry. Ticagrelor (alone and in combination with ASA), ruciromab, and PGE1, but not ASA, prolonged the lag phase and decreased the maximum rate of plasma clotting and decreased the peak and maximum rate of thrombin generation. Inhibition was observed when platelets were not treated with exogenous agonists (activation by endogenous thrombin) and pretreated with collagen. Ticagrelor (alone and in combination with ASA), ruciromab, and PGE1, but not ASA, decreased PS exposure on washed platelets activated by thrombin and by thrombin + collagen. PS exposure on activated platelets in whole blood was lower in patients with acute coronary syndrome receiving ticagrelor + ASA in comparison with donors free of medications. These results indicate that antiplatelet drugs are able to suppress platelet coagulation activity not only in vitro but also after administration to patients.

## 1. Introduction

Platelets are involved in blood coagulation by activation-dependent exposure of negatively charged phosphatidylserine (PS), which serves as a substrate for assembling coagulating complexes [1,2,3,4]. Platelets are able to accelerate thrombin generation and fibrin formation, two final reactions of coagulation cascade [1,2,3,4,5]. 

Antiplatelet drugs or antiaggregants are widely used for prevention and treatment of thrombotic diseases, including myocardial infarction, unstable angina (acute coronary syndrome, ACS), and ischemic stroke. Antiplatelet drugs are divided into several classes depending on the mechanism of their action: (1) an inhibitor of thromboxane A2 (TXA2) synthesis (acetylsalicylic acid, ASA) [6], (2) antagonists of P2Y12 ADP receptors (clopidogrel, prasugrel, and ticagrelor) [7], (3) antagonists (blockers) of platelet fibrinogen receptor, glycoprotein (GP) IIb-IIIa, (abciximab, eptifibatide, tirofiban, and ruciromab (Monafram^®^, Framon Ltd. Moscow, Russia, used in the Russian Federation only)) [8,9,10]. Efficacy of these drugs is routinely assessed by their ability to suppress platelet aggregation. ASA and P2Y12 antagonists effectively inhibit platelet aggregation induced by arachidonic acid (precursor of platelet TXA2) and by ADP, respectively, and produce milder effects on aggregation induced by strong agonists such as thrombin or TRAP (thrombin receptor activating peptide) and collagen [6,7]. GP IIb-IIIa antagonists block the final reaction in platelet aggregation, the formation of fibrinogen bridges between aggregating platelets, thus completely inhibiting all types of aggregation response [8,9,10]. Prostaglandin E1 (PGE1) and prostacyclin (activators of adenylate cyclase), used primarily as vasodilating agents, also effectively inhibit platelet activation and aggregation [11]. 

The effects of all antiplatelet drugs on platelet aggregation has been systematically investigated in numerous experimental and clinical studies [6,7,8,9,10,11]. However, their effects on platelet coagulation activity are not thoroughly explored, and sometimes quite contradictory results are reported. It is still unclear how antiplatelet drugs influence platelet-dependent fibrin formation. There is evidence that antiplatelet drugs are able to inhibit thrombin generation in the presence of platelets [12,13,14,15,16]. However, for ASA and P2Y12 antagonists, this effect was observed predominantly in the presence of specific agonists (arachidonic acid and ADP, respectively) [12,13,14] and depended on the presence and concentration of activated factor VII [13]. PS exposure on activated platelets was partially inhibited by P2Y12 receptor antagonists [17,18], while for GP IIb-IIIa antagonists and ASA, results were rather inconsistent. For different GP IIb-IIIa antagonists, inhibitory effects [16,19,20,21], absence of the effects [22], and even paradox stimulatory [20,21,23] effects were demonstrated. ASA has no effects on PS exposure in vitro [18] but was effective upon its administration to patients [24]. It should also be noted that no systematic studies have been carried out on the simultaneous comparison of the action of antiplatelet drugs with a different mechanism of action on different platelet procoagulant reactions.

In this study, we explored the effects of all types of antiplatelet drugs (ASA, ticagrelor, GP II-IIIa antagonist, and PGE1) on platelet-dependent thrombin generation, on fibrin formation, and on the ability of activated platelets to expose PS.

## 2. Materials and Methods

### 2.1. Donors and Patients

Blood for the preparation of washed platelets (plasma recalcification assay, thrombin generation test (TGT), and PS exposure in washed platelets) was collected from healthy volunteers free of any medication for at least two weeks before blood donation. For evaluation of the effects of antiplatelet drugs on PS exposure in whole blood upon their administration to patients, blood was collected from healthy volunteers and patients with ACS receiving dual antiplatelet therapy, ASA + ticagrelor. Characteristics of both groups are summarized in Table 1. All patients were treated at the Chazov National Medical Research Center of Cardiology. All participants signed informed consent, and the study was approved by the local ethics committee of the Chazov National Medical Research Center of Cardiology (protocol # 274, 9 November 2021).

### 2.2. Blood Collection and Platelet Preparation

For preparation of washed platelets, blood was collected in acid citrate dextrose (ACD, 65 mM citric acid, 85 mM sodium citrate, and 2% dextrose) at a 6/1 blood/anticoagulant ratio. For the measurement of PS exposure in whole blood and platelet aggregation in platelet rich plasma (PRP), blood was collected in 3.8% sodium citrate at a 9/1 blood/anticoagulant ratio. 

Platelet count in whole blood, in PRP, and in suspension of washed platelets (see below) was determined in an Abacus Junior B hematological analyzer (Diatron Ltd. Budapest, Hungary).

PRP was prepared by centrifugation of citrate-anticoagulated blood for 180 g for 10 min.

Washed platelets were prepared from ACD anticoagulated blood as described elsewhere [25] and resuspended in Tyrode/HEPES solution (137 mM NaCl, 2.7 mM KCl, 0.36 mM NaH_2_PO_4_, 0.1% dextrose, 2 mM CaCl_2_, 1 mM MgCl_2_, 0.35% BSA, 5 mM HEPES, pH 7.35) at 0.5 × 10^8^/mL. 

### 2.3. Platelet Aggregation

All antiplatelet agents used in the study were tested for their ability to inhibit platelet aggregation. Platelet aggregation in PRP was recorded using the standard turbidimetric method in a BIOLA aggregation analyzer (BIOLA, Moscow, Russia) as described previously [26]. PRP was not treated with platelet inhibitors (control) or treated with 0.2 mM ASA (Sigma-Aldrich, St. Louis, MO, USA), 1 µM ticagrelor (Sigma-Aldrich, MO), 20 µg/mL ruciromab (F(ab)2 fragment of ant-GP IIb-IIIa monoclonal antibody FRaMon (CRC64)) (Framon Ltd., Moscow, Russia), or 1 µg/mL PGE1 (Sigma-Aldrich, MO) for 5 min at 37 °C. For ASA testing, aggregation was induced by 1 mM arachidonic acid (Santa Cruz Biotechnology, Heidelberg, Germany), for ticagrelor testing, by 20 µM ADP (AppliChem GmbH, Darmstadt, Germany), and for ruciromab and PGE1 testing, by 20 µM TRAP (sequence SFLLRN, provided by Dr. M.D. Ovchinnikov, Chazov National Medical Research Center of Cardiology). All test antiplatelet agents effectively inhibited platelet aggregation induced by the corresponding agonists. ASA completely inhibited aggregation induced by arachidonic acid, ticagrelor inhibited by about 70% aggregation induced by ADP, and ruciromab and PGE1 completely inhibited aggregation induced by TRAP (Supplementary Figure S1. Inhibition of platelet aggregation in PRP by antiplatelet drugs).

### 2.4. Plasma Recalcification Assay

Plasma recalcification assay in the presence of platelets was performed essentially as described previously [4]. Washed platelets at 0.5 × 10^8^/mL were incubated for 5 min at room temperature without any additions (control) or in the presence 0.2 mM ASA, 1 µM ticagrelor, 0.2 mM ASA + 1 µM ticagrelor, 20 µg/mL ruciromab, or 1 µg/mL PGE1. After incubation, platelets were not activated or activated by 10 µg/mL collagen (Revohem, Sysmex, Milton Keynes, UK) for 5 min at room temperature. Platelets (100 µL, 0.5 × 10^7^ per well) were added to 96-well cell-culture flat-bottom plates (Costar®, Corning, Glendale, AZ) and sedimented for 5 min at 1500 g. After removal of the supernatant from sedimented platelets, 50 µL CaCl_2_-free Tyrode/HEPES solution supplemented with 150 μg/mL corn trypsin inhibitor (provided by Dr. G.V. Shekhvatova, Institute of Protein, Pushchino, Russia) for partial inhibition of contact activation and 50 µL citrated plasma (pooled plasma from 3–4 donors depleted of endogenous microparticles by centrifugation for 90 min at 20,000× *g*) were added into the wells. Samples without platelets were also evaluated. Plasma was recalcified by adding 50 μL 25 mM CaCl2 (Diagnostica Stago, Asnières sur Seine, France). Fibrin formation (plasma clotting) was evaluated by changes in light absorbance at 450 nm (A450) for 60 min at 25 °C in a Thermo Scientific Multiscan Go plate spectrophotometer (Thermo Fisher Scientific, Vantaa, Finland). The lag phase (time to reach 5% maximum A450 increase, min) and the maximum rate of fibrin formation (Vmax, maximum increase in A450 per min as percentage of total increase after 60 min (%A450/min)) were determined. 

### 2.5. Thrombin Generation Test

A thrombin generation test (TGT) in the presence of platelets was performed as described earlier [4]. Platelets were prepared and sedimented on the bottom of multiwell plates in the same way as for the plasma recalcification assay (see above). All reagents used in TGT were from Diagnostica Stago (Asnières sur Seine, France). After removal of the supernatant from sedimented platelets, 80 µL citrated plasma and 20 µL trigger PRP reagent (tissue factor (TF) and a minimum amount of phospholipids) were added into the wells and incubated for 10 min at 37 °C. Samples without platelets were also evaluated. The reaction was started by 20 µL Fluo-Buffer (thrombin fluorogenic substrate and CaCl_2_). The final concentration of TF was 0.5 pM. In control samples, thrombin generation was monitored with the use of a “PPP reagent” that provided final concentrations of 5 pM for tissue factor and 4 µM for phospholipids. For calibration of the fluorescent signal, a thrombin calibrator (750 nM Thrombin-α2-macroglobulin complex) was added instead of trigger reagent. Measurements were performed in a Fluoroscan Ascent plate fluorimeter (Thermo Fisher Scientific, Vantaa, Finland). Thrombin generation curves were analyzed using Thrombinoscope 3.0.0.29 software (Thrombinoscope BV, Maastricht, Netherlands). The following parameters were determined: lag phase, endogenous thrombin potential (ETP), peak, and V_max_.

### 2.6. Phosphatidylserine Exposure—Washed Platelets

Phosphatidylserine (PS) exposure on the surface of activated washed platelets was evaluated by flow cytometry using staining with Annexin V-FITC, essentially as described previously [4]. 

Washed platelets (0.5 × 10^8^/mL) were not treated (control) or treated with antiplatelet drugs (see above) and then were not activated or activated for 15 min at 37 °C, without stirring, by 10 U/mL human thrombin (Haematologic Technologies, Inc., Essex Junction, VT, USA) or by thrombin in combination with 10 µg/mL collagen. Platelet suspension (50 µL) was then supplemented with 5 µL annexin V-FITC (BD Biosciences, San Jose, CA, USA) and 3 µL CD42b-APC (BD Pharmingen, San Jose, CA, USA) and incubated for 20 min at room temperature in the dark. Samples without annexin V were used for the gating of PS− platelets. Then, Tyrode/HEPES solution (250 µL) was added, and the samples were analyzed in a FACSCanto II flow cytometer using DivaTM software (BD Biosciences, San Jose, CA). Platelets were gated in according to their size and CD42b-positive staining. The percentage of annexin V-FITC (PS)-positive platelets was calculated in comparison with platelet without annexin V-FITC.

### 2.7. Phosphatidylserine Exposure—Whole Blood

Phosphatidylserine (PS) exposure on the surface of activated platelets was also measured in the whole blood. Blood from healthy volunteers and patients with ACS receiving dual antiplatelet therapy (ASA + ticagrelor) was collected in sodium citrate (see above). In order to prevent blood coagulation after the addition of Ca^+^ containing Tyrode/HEPES solution (calcium was required for effective PS exposure), blood was supplemented with thrombin inhibitor PPACK (D-Phe-Pro-Arg-clormetylketone) (Chinese Peptide Company, Hangzhou, China) at a final concentration of 100 µM. Blood was diluted 20-fold, and platelets were not activated or activated by 20 µM TRAP and by 20 µM TRAP + 10 µg/mL collagen for 15 min at 37 °C without stirring. Then, probes for flow cytometry were prepared and analyzed in the same way as for washed platelets.

### 2.8. Statistics

Statistical analysis was performed using Statistica 12 software (StatSoft. Inc., Tulsa, OK). Most of analyzed variables fit normal distribution (Shapiro–Wilk’s test). Data were expressed as means ± standard deviations (SD). The significance of differences was evaluated using a paired *t*-test or a *t*-test for means (as indicated) or a Chi-square test (for categorical variables).

## 3. Results

### 3.1. Effects of Antiplatelet Drugs on Platelet-Dependent Fibrin Formation

**Figure 1:**
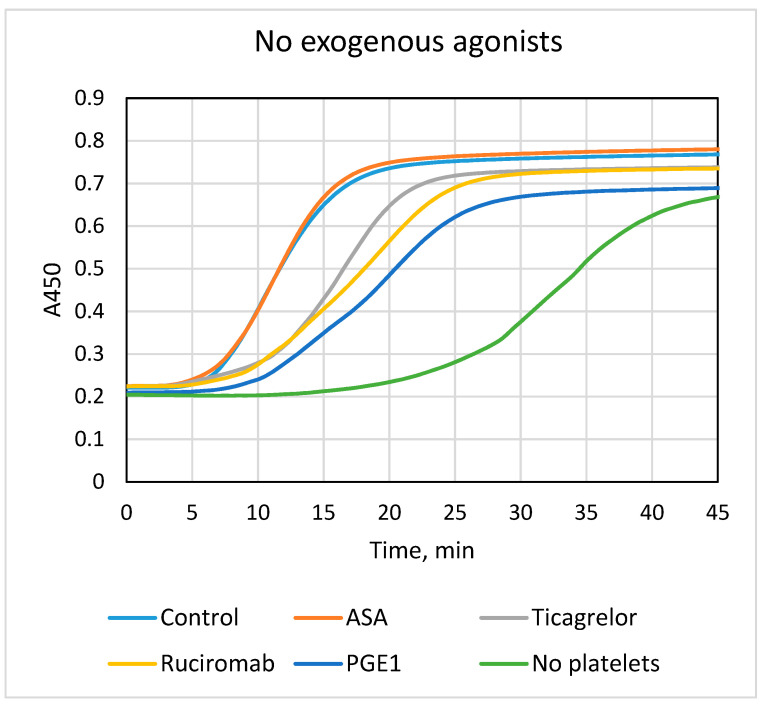

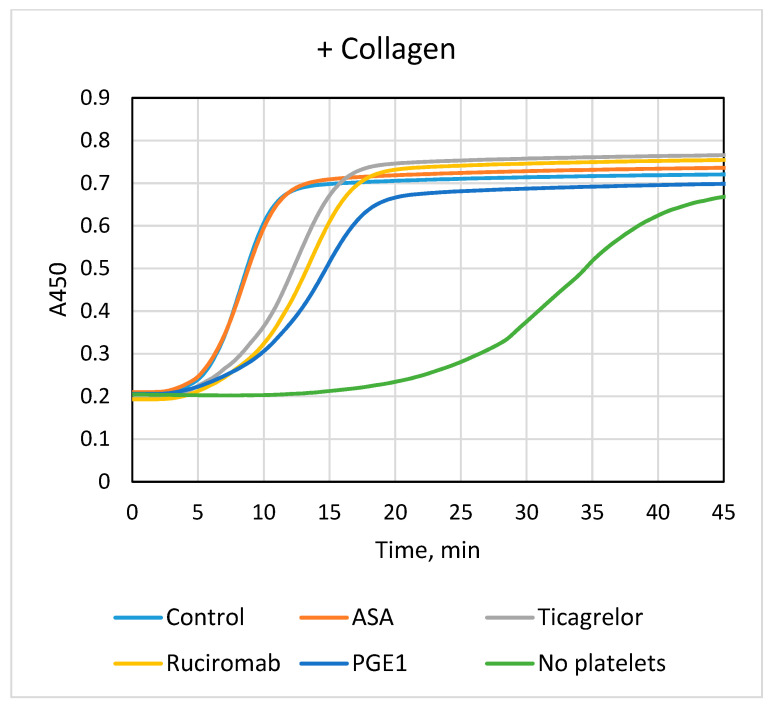
Effects of antiplatelet drugs on fibrin formation (plasma clotting). Plasma recalcification assay was performed in the presence of platelets not treated with antiplatelet drugs (“Control”) or treated with 0.2 mM ASA (“ASA”), 1 µM ticagrelor (Ticagrelor), 20 µg/mL ruciromab, and 1 µg/mL PGE1, or without platelets (“No platelets”). Platelets were not activated with exogenous agonists (upper panel, “No exogenous agonists”) or preactivated with 10 µg/mL collagen (low panel, “+Collagen”). Curves with ticagrelor combined with ASA were the same as with ticagrelor alone and are not shown. Representative results from 6–15 experiments. Statistical data are presented in Table 2.

Fibrin formation in blood plasma (plasma clotting) was evaluated using a modified recalcification assay [4]. Platelets accelerated fibrin formation, shortening the lag phase and increasing Vmax V maxin comparison with platelet-free samples. Platelet activation by collagen in addition to exogenous thrombin formed in plasma provided further acceleration (Figure 1, Table 2). Ticagrelor, ruciromab, and PGE1 partially reduced the rate of fibrin formation. They prolonged the lag phase and decreased the Vmax of plasma clotting. Inhibitory effects were slightly lower for ticagrelor (about 20–30%) than for ruciromab (about 40–50%) and PGE1 (about 40–60%) (all effects were statistically significant). In contrast to other antiplatelet agents, ASA did not slow plasma clotting. ASA also failed to provide any additive effect to the inhibitory action of ticagrelor upon combined application. Similar results were obtained with platelets not treated with exogenous agonists (activation by endogenous thrombin) and with platelets pretreated with collagen (Figure 1, Table 2). 

### 3.2. Effects of Antiplatelet Drugs on Platelet-Dependent Thrombin Generation

**Figure 2:**
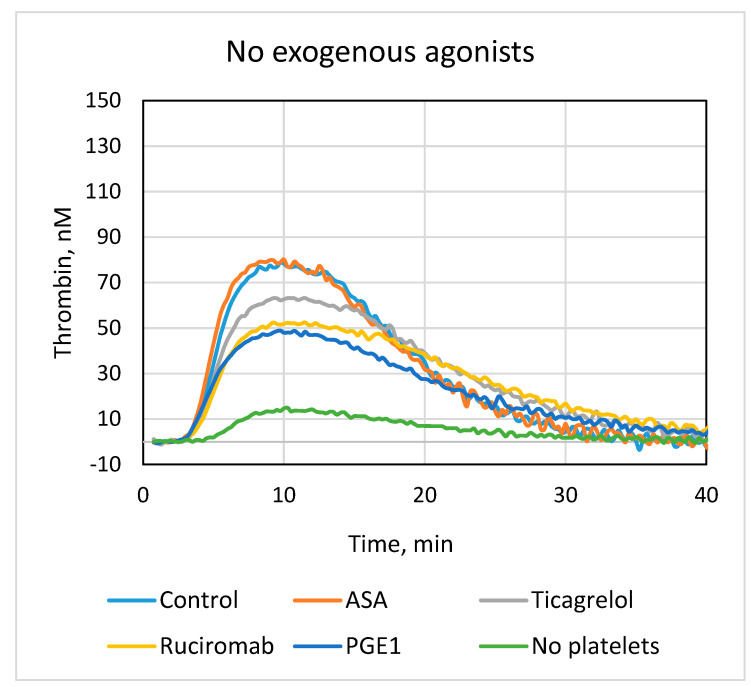

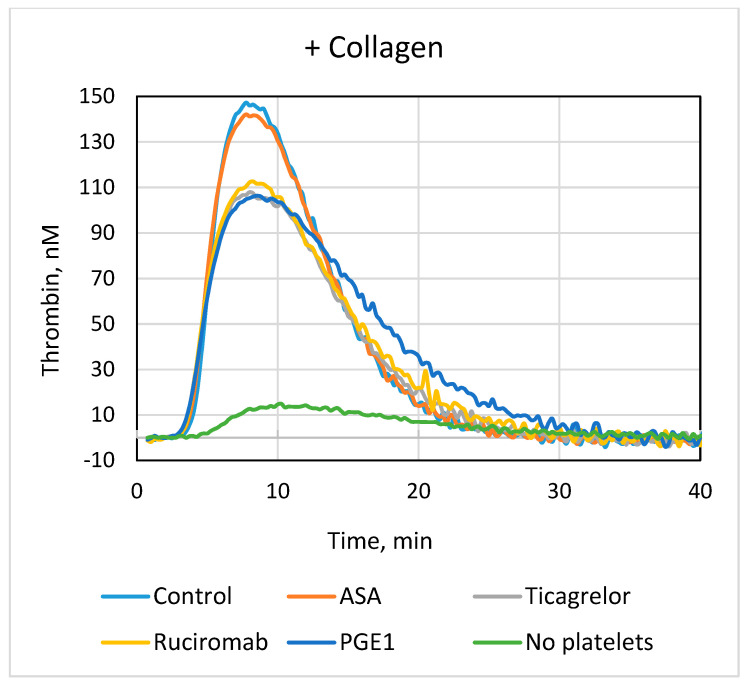
Effects of antiplatelet drugs on thrombin generation. TGT test was performed in the presence of platelets not treated with antiplatelet drugs (“Control”) or treated with 0.2 mM ASA (“ASA”), 1 µM ticagrelor (Ticagrelor), 20 µg/mL ruciromab, and 1 µg/mL PGE1, or without platelets (“No platelets”). Platelets were not activated with exogenous agonists (upper panel, “No exogenous agonists”), or preactivated with 10 µg/mL collagen (lower panel, “+Collagen”). Curves with ticagrelor combined with ASA were the same as with ticagrelor alone and are not shown. Representative results from 5–9 experiments. Statistical data are presented in Table 3.

Thrombin generation in blood plasma was measured using the TGT protocol adapted to assess platelet contribution (low TF and minimum phospholipids). Platelets were prepared in the same way as for recalcification assay. Platelets considerably increased thrombin generation in comparison with platelet-free samples, the stimulatory effect being enhanced after their activation with collagen (Figure 2, Table 3). Ticagrelor, ruciromab, and PGE1 significantly decreased the peak (by about 20–30%) and Vmax (by about 30–40%) of thrombin generation (all effects were statistically significant) and produced negligible effect on the lag phase and the ETP (only PGE1 slightly shortened the lag phase and decreased the ETP in samples without collagen). Thus, these antiplatelet drugs inhibited the primarily platelet-dependent acceleration of thrombin generation but did not affect the duration of the initiation phase (presumably depended on TF) and the total amount of generated thrombin. Again, as in a recalcification assay, ASA did not change any parameters of thrombin generation and failed to potentiate the effects of ticagrelor. Similar results were obtained with platelets not treated with exogenous agonists (activation by endogenous thrombin) and with platelets pretreated with collagen (Figure 2, Table 3). 

### 3.3. Effects of Antiplatelet Drugs on PS Exposure

**Figure 3:**
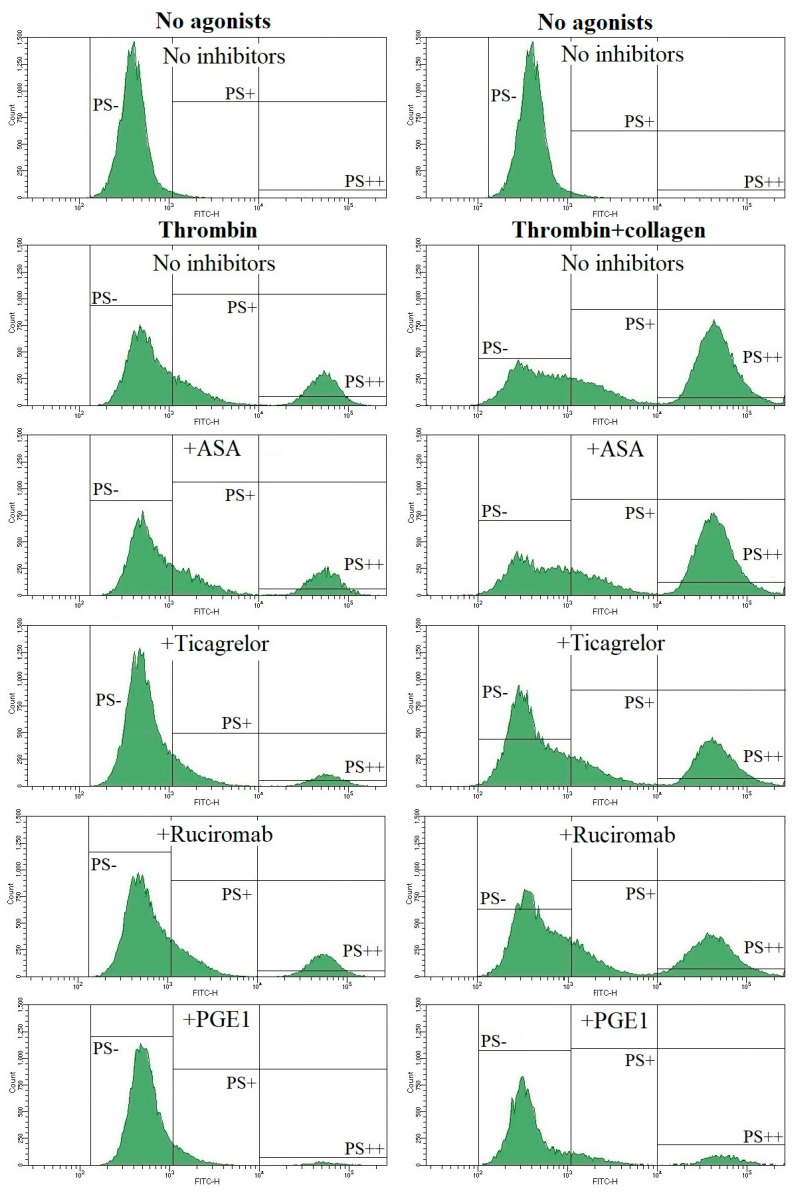
Effects of antiplatelet drugs on PS exposure. Washed platelets. Flow cytometry. Annexin V-FITC is used as PS marker. Vertical lines—borders between PS− (95% of the peak without annexin V-FITC, not shown), PS+, and PS++ regions (the latter as a part of PS+ region). Platelets were not activated (“No agonists”) or were activated with 10 U/mL thrombin (“Thrombin”) or with 10 U/mL thrombin + 10 µg/mL collagen (“Thrombin + collagen”). Platelets were not treated with antiplatelet drugs (“No inhibitors”) or treated with 0.2 mM ASA (“+ASA”), 1 µM ticagrelor (“+Ticagrelor”), 20 µg/mL ruciromab (“+Ruciromab”), and 1 µg/mL PGE1 (“+PGE1”). Histograms with ticagrelor combined with ASA were the same as with ticagrelor alone and are not shown. Representative histograms from 6 experiments. Statistical data are presented in Figure 4.

**Figure 4:**
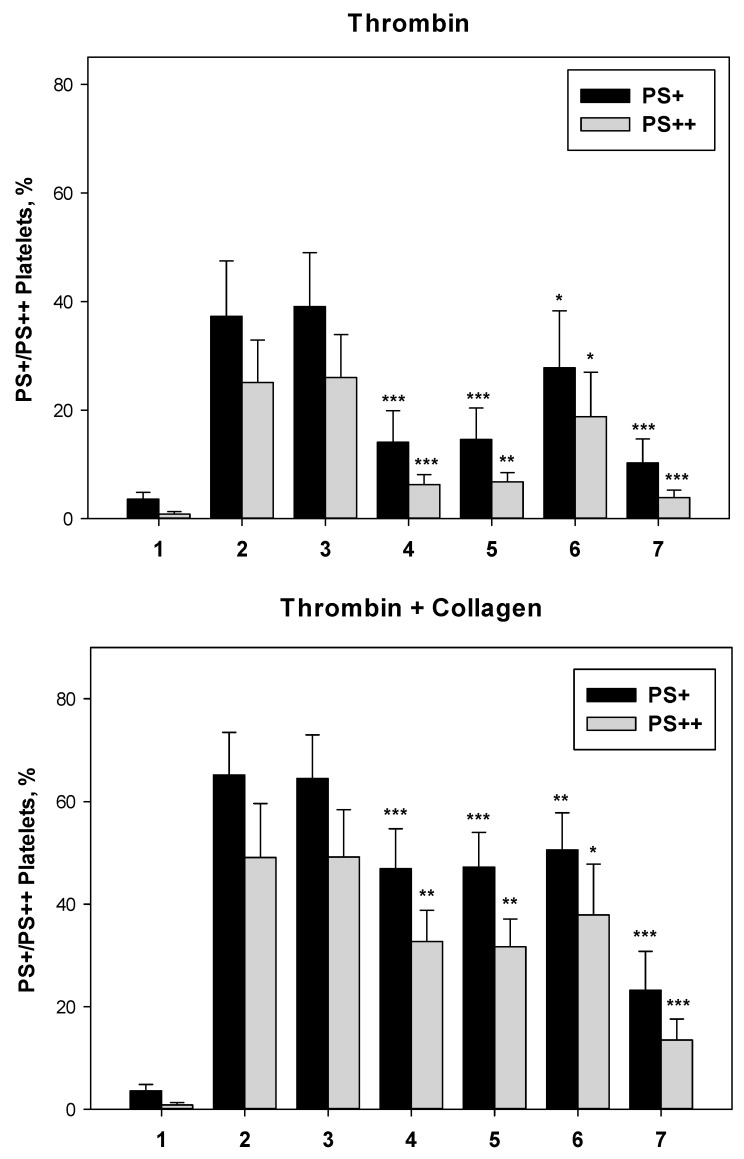
Effects of antiplatelet drugs on PS exposure. Washed platelets. Platelets were not treated with antiplatelet drugs (controls 1 and 2) or treated with 0.2 mM ASA (3), 1 µM ticagrelor (4), 0.2 mM ASA + 1 µM ticagrelor (5), 20 µg/mL ruciromab (6), and 1 µg/mL PGE1 (7). Platelets were not activated (1) or were activated with 10 U/mL thrombin (upper panel, “Thrombin”) or with 10 U/mL thrombin + 10 µg/mL collagen (lower panel, “Thrombin + Collagen). PS exposure was evaluated using flow cytometry (see Figure 3). Means ± SD for the percentage of PS+ (black columns) and PS++ (grey columns) platelets (a subfraction of PS+ platelets) are presented (n = 6). * *p* < 0.05, ** *p* < 0.01, *** *p* < 0.001—Significance of differences from probes without antiplatelet drugs and with corresponding agonists (control 2) (paired *t*-test).

Phosphatidylserine (PS) exposure on the surface of activated platelets was measured by flow cytometry using annexin V as a specific marker. Washed platelets were activated by thrombin alone or in combination with collagen. On histograms, we identified three regions: PS−, platelets not exposing PS, PS+, all platelets exposing PS, and PS++, platelets with a high level of PS exposure, as a subfraction of PS+ platelets (Figure 3). Ticagrelor, ruciromab, and PGE1 decreased the number of PS+ and PS++ platelets after their activation with thrombin and thrombin + collagen, while ASA was ineffective by itself and did not provide any additive effect in combination with ticagrelor. In this test, PGE1 was the most effective (60–80% inhibition) and the effects of ticagrelor depended on the type of platelet activation (about 30% and 60–70% inhibition when platelets were activated by thrombin and by thrombin + collagen, respectively), and rucirtomab was the least effective (20–25% inhibition). However, the inhibitory effects of ruciromab were statistically significant, as well as the effects of ticagrelor and PGE1 (Figure 3, Figure 4). 

Platelets in the whole blood obtained from healthy volunteers free of any medications and patients with ACS receiving dual antiplatelet therapy (ASA + ticagrelor) were activated by TRAP or TRAP + collagen. TRAP was used instead of thrombin in order to avoid blood coagulation. In whole blood, TRAP less effectively stimulated PS than thrombin added to washed platelets (compare data in Figure 4 and Table 4), which agrees with our previous results obtained for both agonists in washed platelets [4]. PS exposure was significantly lower in patients than in healthy volunteers after platelet activation with TRAP alone and with TRAP + collagen (Table 3), indicating that antiplatelet drugs (ticagrelor in particular) suppress this reaction not only in vitro but also in vivo after their administration to patients.

## 4. Discussion

We studied the effects of antiplatelet drugs on platelet-dependent fibrin formation (in plasma recalcification assay) and thrombin generation (in TGT) and on PS exposure on the surface of activated platelets. Several antiplatelet drugs with different mechanisms of action have been tested: ASA, inhibitor of TXA2 synthesis, ticagrelor, antagonist of P2Y12 ADP receptor, ruciromab, GP IIb-IIIa blocker, and PGE1, adenylate cyclase activator. The combination of ASA with ticagrelor was also tested, since this type of dual antiplatelet therapy is commonly used in cardiovascular patients, particularly in ACS patients.

In plasma recalcification assay and in TGT, we used platelets sedimented on the bottom of plastic multiwells. It was shown in our previous study [4] that platelets under applied conditions (see “Materials and Methods” for details) formed a monolayer on a plastic surface. Testing of sedimented platelets, but not platelets in suspension, prevented the effect of platelet aggregation on their coagulation activity, and removal of the supernatant allowed us to avoid the effects of procoagulant microparticles formed by activated platelets. It was reported that both platelet aggregation and formation of microparticles affect platelet-dependent thrombin generation [27].

Thrombin and collagen are the most powerful platelet agonists. At the sites of vascular injury, thrombin is formed from activated prothrombin because of TF-dependent initiation of the coagulation cascade reactions, and subendothelial collagen from the vessel wall is exposed to the blood because of endothelial cell injury. In a recalcification assay, TGT platelets were activated either by endogenous thrombin formed in blood plasma or by endogenous thrombin and exogenous collagen. PS exposure on the platelet surface was also induced by thrombin (in this case exogenous) or TRAP (in the whole blood) alone or in combination with collagen.

We tested for the first time the effects of different antiplatelet drugs on platelet-accelerated fibrin formation (plasma clotting) using a modified plasma recalcification assay. Ticagrelor (both alone and in combination with ASA), ruciromab, and PGE1, but not ASA, were able to prolong the lag phase and to decrease the maximum rate of fibrin formation. Activation of platelets by exogenous thrombin or thrombin + collagen stimulated TXA2 synthesis in platelets and the release of ADP from their dense granules. The efficacy of ticagrelor but not ASA suggested that platelet-derived ADP but not TXA2 is involved in stimulation of platelet-dependent fibrin formation (at least under applied conditions). Arima et al. [28] tested blood obtained from patients with ischemic heart disease receiving ASA or ASA + clopidogrel and from control subjects free of antiplatelet drugs in a flow system where fibrin thrombus formation was evaluated in recalcified blood stimulated by surface-immobilized TF and collagen. They found no differences between these groups; however, the inefficacy of both drugs can be explained by a high impact of TF in coagulation in their system (diminishing platelet contribution). The inhibitory effects of ruciromab (fragment of anti-IIb-IIIa blocking antibody) on platelet-dependent fibrin formation suggested the involvement of GP IIb-IIIa in this reaction. These results are generally in agreement with the early findings by Goto et al. [20] and Ramström et al. [21], who detected the inhibitory effect of another antibody-derived GP IIb-IIIa blocker, abciximab, (but not of low-molecular-weight antagonists) on spontaneous blood clotting and coalin-induced PRP clotting, respectively, with additional platelet activation in both cases.

In TGT, we used the protocol recommended for the evaluation of platelet effects (low amount of TF and minimum phospholipids). Our results obtained in TGT correlated with the results obtained in the plasma recalcification assay. Ticagrelor (both alone and in combination with ASA), ruciromab, and PGE1, but not ASA, decreased the peak and the maximum rate of thrombin generation. Inhibitory effects on thrombin generation were previously demonstrated for different types of antiplatelet agents [12,13,14,15,16]. However, for ASA and P2Y12 antagonists, these effects were detected primarily in the presence of arachidonic acid, a precursor of TXA2, and ADP, respectively [12,13,14]. We did not add these agonists; nevertheless, TXA2 synthesis and ADP release from dense granules can be stimulated in platelets activated by endogenous thrombin and thrombin + collagen. A decrease in thrombin generation by ticagrelor and the inefficacy of ASA confirmed the results obtained in the recalcification assay, suggesting that platelet-derived ADP but not TXA2 participates in the realization of platelet coagulation activity. Berezovskaya et al. [29] have shown that thrombin generation in PRP in patients with coronary artery disease on dual antiplatelet therapy, ASA + clopidogrel, was lower than in healthy donors. These data indicated that the combination of ASA with P2Y12 antagonist (in this case clopidogrel) could suppress thrombin generation not only in vitro but also in in patients receiving this treatment. Our results with the GP IIb-IIIa blocker ruciromab in TGT confirm the ability of this type of antiplatelet drugs to inhibit thrombin generation, which was earlier demonstrated for another antibody-derived preparation, abciximab [15,16], and for low-molecular-weight preparations (peptide eptifibatide and peptidomimetic tirofiban) [16].

Phosphatidylserine (PS) exposure on the surface of activated platelets is responsible for the platelet-dependent acceleration of coagulation reactions [1,2,3,4]. Testing the effects of antiplatelet agents on PS exposure, we expectedly found that similarly to recalcification assay and TGT, ticagrelor, ruciromab, and PGE1, but not ASA, were able to decrease the level of PS exposure on activated platelets. Kotova et al. [18] also found that in vitro ASA produces no effect on the formation of so-called “coated” platelets positive for PS (and for some other markers). However, Prodan et al. [24] detected significant effects of ASA on the level of “coated” platelets when the drug was taken by patients. This discrepancy between ASA effects in vitro and in vivo has no reasonable explanation and needs to be further investigated. Inhibition of PS exposure by ticagrelor is generally consistent with previous findings [17,18], although with other P2Y12 receptor antagonists. Contradictory evidence was obtained for the effects of GP IIb-IIIa blockers on PS exposure: suppression of PS exposure [16,19,20,21], no effects [22], and even paradox stimulatory effects on this reaction [20,21,23]. However, unexpected stimulation of PS exposure was detected only for low-molecular-weight agents [20,21,23] and not in all papers [16], while results with the antibody-derived preparation abciximab, demonstrating its inhibitory action [19,20,21], are consistent with our data obtained with the antibody-derived preparation ruciromab.

In order to demonstrate that antiplatelet drugs can decrease platelet procoagulant activity not only in in vitro experiments but also upon their administration to patients, we performed a pilot study that demonstrated that PS exposure on activated platelets from ACS patients receiving standard dual antiplatelet therapy—ASA + ticagrelol was lower than in healthy subjects free of antiplatelet drugs. However, further and wider investigations with patients and/or volunteers are required for studying (1) the effects of the separate administration of aspirin on PS exposure and (2) the action of different antiplatelet therapies directly on platelet-dependent coagulation reactions (fibrin formation and thrombin generation).

## 5. Conclusions

Complex investigation of the effects of different antiplatelet drugs on platelet procoagulant activities, including their ability to accelerate fibrin formation (plasma clotting) and thrombin generation, and to expose PS on their surface, has shown in in vitro that all these reactions were suppressed by the antagonist of P2Y12 ADP receptor ticagrelor, the GP IIb-IIIa (fibrinogen receptor) blocker rucirimab, and the adenylate cyclase activator PGE1, but not by ASA, an inhibitor of TXA2 synthesis. Limited clinical study demonstrated that the combination of ASA + ticagrelor reduced PS exposure on activated platelets upon administration to ACS patients.

## Figures and Tables

**Table 1:** Characteristics of healthy volunteers and patients with ACS included in the study for the evaluation of PS exposure in whole blood.

	Healthy Volunteers	Patients with ACS
n	30	26
Age, years, mean ± SD	56 ± 16	63 ± 10
Men/women	18/12	17/9
Diagnosis	-	
Myocardial infarction	-	23
Unstable angina	-	3
Percutaneous coronary intervention	-	26
Antiplatelet drugs	-	ASA 100 mg/daily + ticagrelor 90 mg × 2/daily
Time of blood collection (to the day of ACS onset)	-	4–6 days

Differences in age (*t*-test for means) and men/women ratio (Chi-square test) are not significant.

**Table 2:** Effects of antiplatelet drugs on platelet-dependent fibrin formation in blood plasma (recalcification assay).

No Exogenous Agonists		
	Lag phase, min	Vmax, %A450/min
Control (n = 15)	6.4 ± 1.7	11.8 ± 2.5
ASA (n = 11)	6.1 ± 1.8	12.1 ± 4.3
Ticagrelor (n = 15)	8.0 ± 1.7 ***	7.9 ± 2.3 ***
ASA + Ticagrelor (n = 10)	7.2 ± 1.2 ***	8.6 ± 2.4 **
Ruciromab (n = 7)	9.8 ± 1.5 **	5.7 ± 0.8 ***
PGE1 (n = 7)	10.1 ± 1.0 ***	4.8 ± 0.7 ***
**+Collagen**		
	Lag phase, min	Vmax, % A450/min
Control (n = 14)	5.1 ± 1.2	18.7 ± 3.8
ASA (n = 9)	5.1 ± 1.1	17.8 ± 4.5
Ticagrelor (n = 15)	6.3 ± 1.4 ***	12.6 ± 3.8 ***
ASA + Ticagrelor (n = 8)	6.0 ± 1.1 **	11.8 ± 3.0 **
Ruciromab (n = 6)	7.1 ± 1.7 **	11.4 ± 2.2 **
PGE1 (n = 6)	7.5 ± 1.9 **	10.2 ± 2.3 **

Recalcification assay was performed without exogenous platelet agonists or after pretreatment of platelets with collagen (10 µg/mL). Means ± SD are presented (n—number of experiments). ** *p* < 0.01, *** *p* < 0.001—significance of differences from “Control” (no antiplatelet drugs) (paired *t*-test). Parameters of fibrin formation without platelets: lag phase—23.1 ± 7.0 min, Vmax—6.4 ± 1.2 %A450/min (n = 15).

**Table 3:** Effects of antiplatelet drugs on thrombin generation in blood plasma (TGT).

No Exogenous Agonists				
	Lag phase, min	ETP ^1^, nM × min	Peak, nM	Vmax, nM/min
Control (n = 9)	3.9 ± 0.3	1137 ± 146	84 ± 23	17 ± 7
ASA (n = 5)	3.7 ± 0.1	1177 ± 204	93 ± 28	20 ± 8
Ticagrelor (n = 7)	3.8 ± 0.2	1058 ± 164	60 ± 17 **	10 ± 4 **
ASA + Ticagrelor (n = 5)	3.6 ± 0.3	1103 ± 236	67 ± 26 *	12 ± 6 *
Ruciromab (n = 6)	3.7 ± 0.3	1115 ± 190	62 ± 27 *	12 ± 6 *
PGE1 (n = 5)	3.5 ± 0.3 *	1007 ± 186 *	62 ± 18 *	10 ± 3 *
**+Collagen**				
	Lag phase, min	ETP ^1^, nM × min	Peak, nM	Vmax, nM/min
Control (n = 8)	3.8 ± 0.3	1234 ± 140	137 ± 24	36 ± 10
ASA (n = 7)	3.8 ± 0.2	1257 ± 125	131 ± 15	33 ± 5
Ticagrelor (n = 7)	3.8 ± 0.3	1277 ± 193	109 ± 30 **	25 ± 12 **
ASA + Ticagrelor (n = 7)	3.8 ± 0.3	1284 ± 145	111 ± 21 *	25 ± 6 **
Ruciromab (n = 7)	3.7 ± 0.3	1327 ± 193	115 ± 17 *	26 ± 5 *
PGE1 (n = 7)	3.9 ± 0.2	1222 ± 183	105 ± 24 **	24 ± 8 **

Thrombin generation test (TGT) was performed without exogenous platelet agonists or after pretreatment of platelets with collagen (10 µg/mL). ^1^ ETP—endogenous thrombin potential. Means ± SD are presented (n—number of experiments). * *p* < 0.05, ** *p* < 0.01—significance of differences from “Control” (no antiplatelet drugs) (paired *t*-test). Thrombin generation parameters without platelets: lag phase—4.3 ± 0.3 min, ETP—302 ± 49 nM × min, peak—20 ± 3 nM, V_max_—3.7 ± 0.6 nM/min (n = 9).

**Table 4:** Phosphatidylserine (PS) exposure on platelets from healthy volunteers free of any medications and patients with ACS receiving dual antiplatelet therapy (ASA + ticagrelor). Whole blood.

	Healthy Volunteers (No Medications)		Patients with ACS (ASA + Ticagrelor)	
	PS+ Platelets, %	PS++ Platelets, %	PS+ Platelets, %	PS++ Platelets, %
No agonists	1.9 ± 1.3 (n = 30)	0.4 ± 0.4 (n = 30)	1.2 ± 1.0 (n = 25) *p* = 0.022	0.2 ± 0.2 (n = 25) *p* = 0.014
TRAP	10.9 ± 5.1 (n = 20)	3.1 ± 2.8 (n = 20)	5.1 ± 2.8 (n = 19) *p* < 0.001	1.2 ± 1.3 (n = 19) *p* = 0.011
TRAP + collagen	52.0 ± 7.6 (n = 28)	36.4 ± 10.2 (n = 28)	41.7 ± 10.2 (n = 26) *p* < 0.001	26.6 ± 11.9 (n = 26) *p* = 0.002

Phosphatidylserine (PS) exposure on platelets was evaluated in whole blood. Platelets were not activated (“No agonists”) or activated with TRAP (20 µM) or TRAP (20 µM) + collagen (10 µg/mL). Data for PS+ and PS++ platelets (the latter is a subfraction of PS+ platelets) are shown. Means ± SD are presented (n—number of analyses). *p*—significance of differences from “Healthy volunteers” (*t*-test for means).

## Data Availability

The data presented in this study are available only here in the article and supplementary material.

## Supplementary Materials

The following supporting information can be downloaded at https://www.mdpi.com/article/10.3390/biom13071124/s1: Figure S1. Inhibition of platelet aggregation in PRP by antiplatelet drugs.
