# Supplementary material for: Effects of Antiplatelet Drugs on Platelet-Dependent Coagulation Reactions
Source: Biomolecules. 2023 Jul 14;13(7):1124. doi: 10.3390/biom13071124 (PMC10377112; doi:10.3390/biom13071124)
Supplement: Supplementary file 1 [file biomolecules-13-01124-s001.zip › biomolecules-2478400-supplementary.pdf]

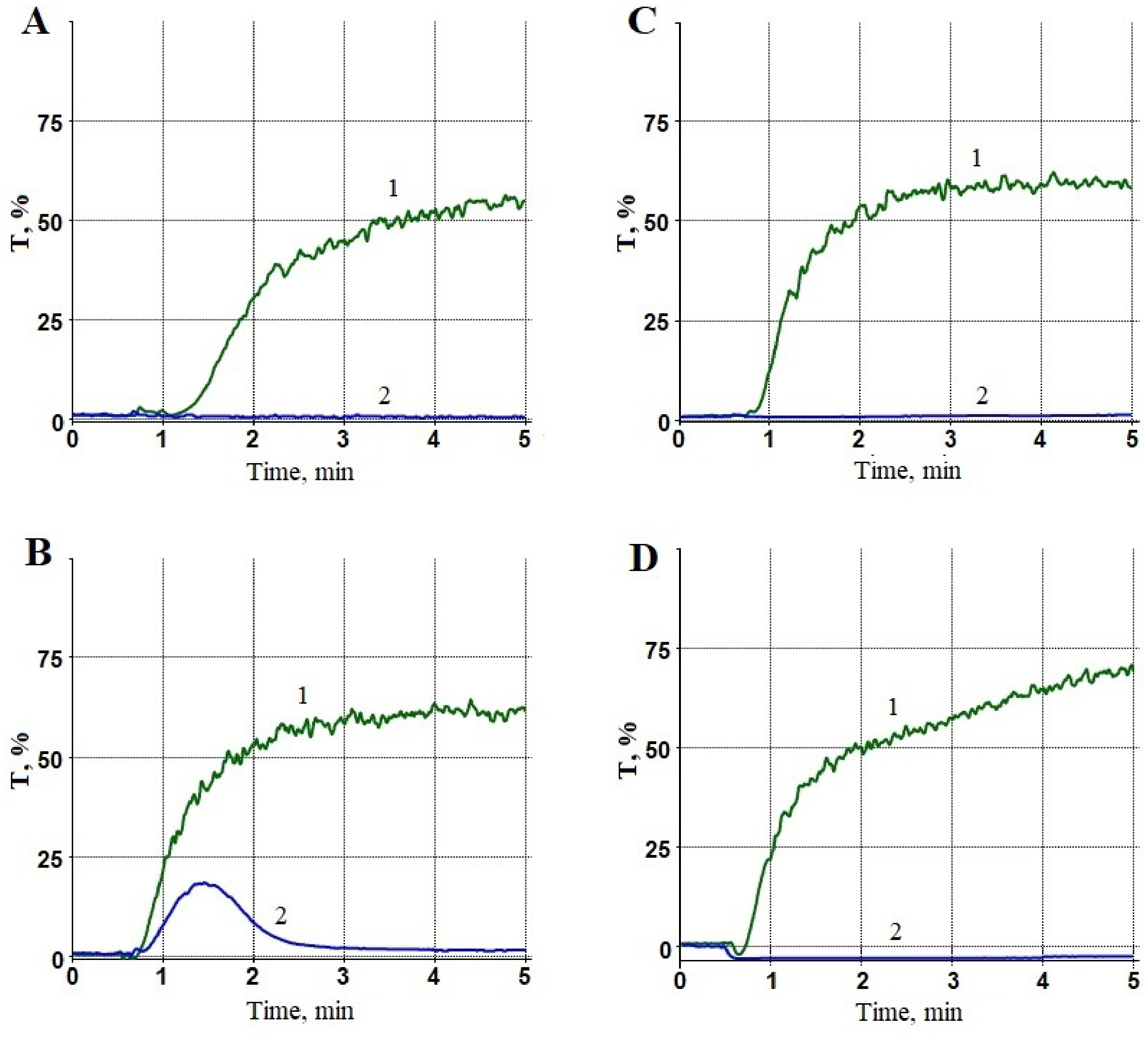

Suppl. Fig. S1. Inhibition of platelet aggregation in PRP by antiplatelet drugs. Light transmission aggregometry. PRP was preuncubated without addition of any drugs (curves 1, green) or in the presence of 0.2 mM ASA (A), 1 µM ticagrelor (B), 1 µg/ml PGE1 (C) or 20 µg/ml ruciromab (D) (curves 2, blue). Platelet aggregation was induced by 1 mM arachidonic acid (A), 10 µM ADP (B), or 20 µM TRAP (C and D). Agonists were added at 30 sec after starting registration.
